# Association Between Metabolic Dysfunction-Associated Fatty Liver Disease and Cardiovascular Risk in Patients With Rheumatoid Arthritis: A Cross-Sectional Study of Chinese Cohort

**DOI:** 10.3389/fcvm.2022.884636

**Published:** 2022-05-13

**Authors:** Yao-Wei Zou, Qian-Hua Li, Jing-Wei Gao, Jie Pan, Jian-Da Ma, Le-Feng Chen, Jian-Zi Lin, Ying-Qian Mo, Xue-Pei Zhang, Pin-Ming Liu, Lie Dai

**Affiliations:** ^1^Department of Rheumatology, Sun Yat-sen Memorial Hospital, Sun Yat-sen University, Guangzhou, China; ^2^Department of Cardiology, Sun Yat-sen Memorial Hospital, Sun Yat-sen University, Guangzhou, China

**Keywords:** cardiovascular disease, rheumatoid arthritis, metabolic dysfunction-associated fatty liver disease, non-alcoholic fatty liver disease, inflammation

## Abstract

**Background:**

The nomenclature from non-alcoholic fatty liver disease (NAFLD) to metabolic dysfunction-associated fatty liver disease (MAFLD) is considered to identify more cardiovascular disease (CVD) risks in the general population. Patients with rheumatoid arthritis (RA) carry an excess risk for CVD. However, the prevalence of MAFLD and its relationship with CVD risks in RA have not been reported.

**Methods:**

This cross-sectional study retrospectively analyzed clinical data from a Chinese RA cohort. MAFLD was diagnosed according to the criteria proposed by an international expert panel from 22 countries in 2020. CVD risk in patients with RA was estimated by the Prediction for Atherosclerotic Cardiovascular Disease Risk in China with a 1.5 multiplication factor.

**Results:**

Among 513 included patients with RA, 78.4% were women and the mean ± SD age was 51.8 ± 12.6 years. The prevalence of MAFLD was 21.4%. There were 10.9% patients with RA concomitated with CVD events and 32.4% with a high-estimated 10-year CVD risk. Besides a higher liver fibrosis score and a higher ratio of advanced fibrosis, RA patients with MAFLD had a higher rate of CVD events (17.3 vs. 9.2%) and a higher proportion of high estimated 10-year CVD risk (55.5 vs. 26.1%) than those without. Multivariate logistic regression analysis showed that MAFLD was associated with an increase in CVD events [adjusted odds ratio (AOR) = 2.190, 95% CI 1.135–4.227] and high estimated 10-year CVD risk (AOR = 2.483, 95% CI 1.412–4.365, all *p* < 0.05).

**Conclusion:**

Metabolic dysfunction-associated fatty liver disease was associated with increased CVD risk in patients with RA, which implies the importance of early detection and management of MAFLD in patients with RA.

## Introduction

Rheumatoid arthritis (RA) is a progressive autoimmune disease characterized by persistent joint inflammation that causes joint damage, deformities, and dysfunction. As a systemic inflammatory disease, extra-articular organ involvement is common in RA, such as cardiovascular, pulmonary, neurological, gastrointestinal, renal, and hematologic diseases (1). Since inflammation acts a central role in the development of cardiovascular disease (CVD), it is not surprising that patients with RA have a higher risk of cardiovascular morbidity and mortality (e.g., myocardial infarction, stroke, heart failure, etc.) (2, 3). In general, about 50% of deaths among patients with RA are attributable to CVD-related causes (3). CVD mortality is increased by approximately 50% in patients with RA compared with the general population (4). In addition to traditional CVD risk factors, RA disease characteristics may play the roles in the development of CVD among patients with RA. Thus, it is of great importance to identify the strong predictors of CVD and take an early intervention in RA.

Non-alcoholic fatty liver disease (NAFLD) has emerged as the most common chronic liver disease worldwide (5). NAFLD may progress to fibrosis, cirrhosis, and hepatoma, resulting in a raised hazard for metabolic and CVD deaths (5, 6). Metabolic dysfunction-associated fatty liver disease (MAFLD), a novel nomenclature changing from NAFLD in 2020, has its inclusion criteria by adding overweight or obesity, type 2 diabetes mellitus (T2DM), and evidence of metabolic dysregulation based on the definitions of NAFLD (7). The alteration from NAFLD to MAFLD was proposed to identify metabolic fatty liver regardless of alcohol intake or other concomitant liver diseases (7). It has been suggested that the NAFLD-to-MAFLD alteration can recognize higher CVD risks in the general population (8).

However, the prevalence of MAFLD and its relationship with CVD risks in patients with RA have not been reported. We investigated the prevalence of MAFLD and conducted a cross-sectional analysis of its relationship with CVD risks in patients with RA.

## Materials and Methods

### Study Design and Participants

This retrospective study was carried out based on our RA cohort (9–12) at the Department of Rheumatology, Sun Yat-sen Memorial Hospital, China. Subjects more than 16 years old with a confirmed diagnosis of RA (2010 criteria) (13) were recruited when they finished abdominal ultrasound examination from June 2015 to September 2021. Subjects with other autoimmune diseases, serious infections, malignancy, and pregnancy were excluded. Ethical approval mandatory for this study was obtained from Ethics Committee at Sun Yat-sen Memorial Hospital (SYSEC-KY-KS-2020-208) along with informed consent from each patient.

### Data Collection

Available demographic and clinical data were collected at enrollment as we previously reported (9–12). RA disease activity was assessed using the clinical disease activity index (CDAI), simplified disease activity index (SDAI), disease activity score in 28 joints with four variables including CRP (DAS28-CRP), and disease activity score in 28 joints with four variables including ESR (DAS28-ESR). Physical function was assessed with the Stanford Health Assessment Questionnaire Disability Index (HAQ-DI). Conventional radiographs of bilateral hands and wrists (anteroposterior view) were assessed with the Sharp/van der Heijde modified score.

### Laboratory Measurement and Index Calculation

Overnight fasting venous blood samples were collected. Liver biochemistry parameters and metabolic parameters [including alanine aminotransferase (ALT), aspartate aminotransferase (AST), γ-glutamyl transferase (GGT), alkaline phosphatase (ALP), total bilirubin (TBIL), albumin (ALB), fasting plasma glucose (FPG), glycated hemoglobin (HbA1c), fasting insulin (FINS), total cholesterol (TC), triglyceride (TG), high-density lipoprotein cholesterol (HDL-C), and low-density lipoprotein cholesterol (LDL-C)] were measured. Abnormal liver function tests (LFTs) were defined as at least 1 value higher than the upper limit of normal (ULN) for ALT, AST, GGT, ALP, and/or TBIL (14). The homeostasis model assessment of insulin resistance (HOMA-IR) was calculated to evaluate insulin resistance (IR) (15). The fatty liver index (FLI) and non-invasive liver fibrosis models [including fibrosis-4 index (FIB-4), NAFLD fibrosis score (NFS), and Forns index] were evaluated by original formulas (5, 16). Advanced fibrosis was defined by FIB-4 ≥1.3 (age <65 years) or ≥2.0 (age ≥65 years), NFS ≥-1.455 (age <65 years) or ≥0.12 (age ≥65 years), and Forns index ≥ highest quartiles (5, 7, 16).

### Assessment of Metabolic Dysfunction-Associated Fatty Liver Disease

Hepatic steatosis was defined by abdominal ultrasound examination. MAFLD was diagnosed if a subject had hepatic steatosis with one or more of the three criteria: overweight or obese [body mass index (BMI) ≥23 kg/m^2^; by the Asia-Pacific criteria], type 2 diabetes mellitus (T2DM), and metabolic abnormalities described by at least any two indicators: (1) waist circumference (WC) ≥90 cm in men and ≥80 cm women; (2) blood pressure (BP) ≥130/85 mmHg or taking anti-hypertension drugs; (3) TG ≥150 mg/dl or taking lipid-lowering agents; (4) HLD-C < 40 mg/dl in men and <50 mg/dl in women or taking lipid-lowering agents; (5) prediabetes (e.g., FPG 100 to 125 mg/dl or 2-h post-load glucose levels 140 to 199 mg/dl or HbA1c 5.7 to 6.4%; (6) HOMA-IR ≥2.5; and (7) high-sensitivity C-reactive protein (hs-CRP) level >2 mg/L (7). In this study, we had no data on hs-CRP. CRP (scatter turbidimetry method, Siemens Healthcare Diagnostics, range: 0–5 mg/L) was used instead of hs-CRP, because CRP was recommended as a disease activity indicator by the American College of Rheumatology (ACR) and European League Against Rheumatism (EULAR) guideline for RA management (13), and the elevated CRP was defined as CRP >5 mg/L.

### Cardiovascular Disease Events and 10-Year Cardiovascular Disease Risk Estimation

Cardiovascular disease events were defined as a verified medical history of coronary, cerebral, and peripheral arterial disease, including angina pectoris, heart failure, myocardial infarction, ischemic or hemorrhagic stroke, and peripheral arterial disease (3). CVD events were collected by a questionnaire survey combined with verification through medical records.

The estimated 10-year CVD risk was assessed by the Prediction for Atherosclerotic Cardiovascular Disease (ASCVD) Risk in China (China-PAR) (17), Systemic Coronary Risk Evaluation (SCORE) for the low- and high-risk regions in Europe (18), and Framingham Risk Score (FRS) (19). The 10-year CVD risk estimation for patients with RA was performed using a 1.5 multiplication factor to the risk estimated by the CVD risk calculator used in the general population according to the EULAR recommendation (3) and was classified into low, moderate, and high risk according to the Chinese guidelines on the primary prevention of cardiovascular diseases (20).

### Statistical Analysis

Patient characteristics were summarized as mean ± standard deviations (SD), medians (interquartile range [IQR]), or frequencies (%) where applicable. We used the *t*-test, the Mann–Whitney *U* test, the chi-square test, or Fisher’s exact tests when appropriate, to determine the differences between groups. The estimated 10-year CVD risk was transformed into dichotomous variables (low-moderate-risk and high-risk). Logistic regression modeling was conducted to examine the estimated odds ratio (OR) for the MAFLD in relation to the risk of CVD events and the high estimated 10-year CVD risk. Statistical Package for the Social sciences (SPSS) and R statistical software were used for all analyses. A two-sided *p*-value of <0.05 was considered statistically significant.

## Results

### Demographic and Clinical Characteristics of Patients With Rheumatoid Arthritis

Among 755 enrolled patients with RA, 242 who had missing laboratory data or abdominal ultrasound examinations were excluded. Thus, a total of 513 patients with RA were qualified for statistical analysis. As shown in Table 1, 78.4% included patients with RA were women, with a mean age of 51.8 ± 12.6 years. The median disease duration was 60 months (IQR 21 to 120 months). According to CDAI, 85.4% patients with RA were active (CDAI >2.8), whereas 14.6% were in remission (CDAI ≤2.8). Moreover, 27.5% patients with RA have not received any previous glucocorticoid or disease-modifying anti-rheumatic drug (DMARD) therapy for 6 months before enrollment (treatment naïve).

**TABLE 1:** Demographic and clinical characteristics of RA patients with MAFLD.

Characteristics	Patients with RA (*n* = 513)	No-MAFLD (*n* = 403)	MAFLD (*n* = 110)	*P*
Age, years, mean ± SD	51.8 ± 12.6	51.1 ± 13.2	54.4 ± 9.5	0.027
Female, *n* (%)	402 (78.4)	317 (78.7)	85 (77.3)	0.754
Active smoking, *n* (%)	90 (17.5)	68 (16.9)	22 (20.0)	0.445
Disease duration, months, median (IQR)	60 (21,120)	57 (20,120)	60 (24,132)	0.415
Positive RF, *n* (%)	366 (71.3)	287 (71.2)	79 (71.8)	0.901
Positive ACPA, *n* (%)	370 (72.1)	289 (71.7)	81 (73.6)	0.690
**Disease activity indicators**				
28TJC, median (IQR)	4 (1,9)	4 (1,10)	4 (1,9)	0.821
28SJC, median (IQR)	2 (0,7)	2 (0,7)	2 (0,7)	0.733
PtGA, cm, median (IQR)	4 (2,6)	4 (2,6)	5 (3,6)	0.122
PrGA, cm, median (IQR)	4 (2,6)	4 (2,6)	4 (2,6)	0.123
Pain VAS, cm, median (IQR)	4 (2,5)	4 (2,5)	4 (2,5)	0.258
ESR, mm/h, median (IQR)	39 (21,70)	40 (20,70)	37 (21,66)	0.744
CRP, mg/L, median (IQR)	7.7 (3.3,30.4)	8.3 (3.3,30.6)	6.4 (3.3,27.4)	0.915
CDAI, median (IQR)	16 (6,28)	14 (6,28)	16 (8,26)	0.485
SDAI, median (IQR)	17.3 (7.3,31.9)	17.1 (6.3,31.5)	17.9 (8.5,32.6)	0.516
DAS28-CRP, median (IQR)	4.0 (2.8,5.4)	3.9 (2.6,5.4)	4.0 (3.1,5.4)	0.467
DAS28-ESR, median (IQR)	4.7 (3.2,6.1)	4.7 (3.0,6.2)	4.6 (3.4,6.0)	0.914
**Functional indicator**				
HAQ-DI, median (IQR)	0.50 (0.00,1.13)	0.38 (0.00,1.25)	0.62 (0.13,1.13)	0.222
**Radiographic indicators**				
mTSS, median (IQR)	9 (1,34)	10 (1,34)	9 (2,33)	0.657
JSN, median (IQR)	2 (0,15)	2 (0,16)	1 (0,13)	0.129
JE, median (IQR)	6 (0,19)	6 (0,19)	7 (1,19)	0.796
**Previous medications**				
Treatment naïve^Δ^, *n* (%)	141 (27.5)	107 (26.6)	34 (30.9)	0.364
Glucocorticoid, *n* (%)	262 (51.1)	202 (50.1)	60 (54.5)	0.411
csDMARDs, *n* (%)	322 (62.8)	258 (64.0)	64 (58.2)	0.262
Biologic agents, *n* (%)	42 (8.2)	34 (8.4)	8 (7.3)	0.693

*^Δ^Treatment naïve, without previous corticosteroids or DMARDs treatment for 6 months before recruited.*

*MAFLD, metabolic dysfunction-associated fatty liver disease; RF, rheumatoid factor; ACPA, anti-cyclic citrullinated peptide antibody; 28TJC, 28-joint tender joint counts; 28SJC, 28-joint swollen joint counts; PtGA, patient global assessment of disease activity; PrGA, provider global assessment of disease activity; Pain VAS, pain visual analog scale; ESR, erythrocyte sedimentation rate; CRP, C-reactive protein; CDAI, clinical disease activity index; SDAI, simplified disease activity index; DAS28-CRP, disease activity score in 28 joints with four variables including CRP; DAS28-ESR, disease activity score in 28 joints with four variables including ESR; HAQ-DI, health assessment questionnaire disability index; mTSS, modified total Sharp score; JE, joint erosion; JSN, joint space narrowing; csDMARDs, conventional synthetic disease-modifying anti-rheumatic drugs; SD, standard deviations; IQR, interquartile range.*

### Prevalence of Metabolic Dysfunction-Associated Fatty Liver Disease in Patients With Rheumatoid Arthritis With Different Stratification

**FIGURE 1:**
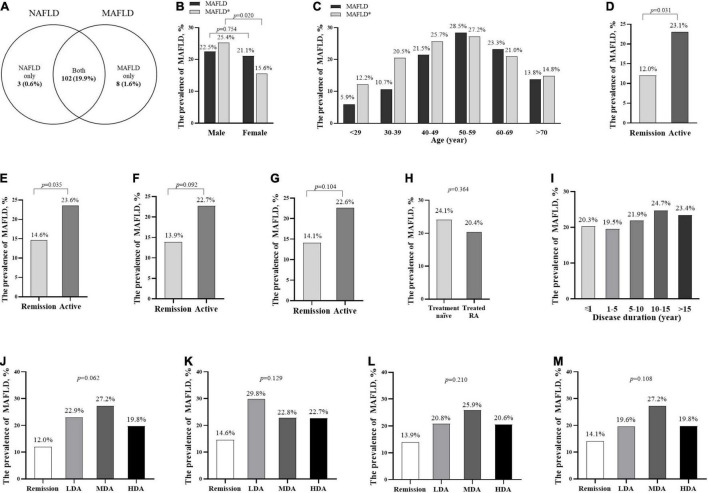
The prevalence of MAFLD in patients with RA with different stratification. The prevalence of MAFLD and NAFLD in all patients with RA **(A)** the prevalence and standardized prevalence in different genders **(B)** and age groups **(C)**; the prevalence in different disease activity groups according to CDAI **(D,J)**, DAS28-CRP **(E,K)**, SDAI **(F,L)**, DAS28-ESR **(G,M)**, treatment groups **(H)**, and disease duration groups **(I)**. *The standardized prevalence of MAFLD was adjusted the age or sex composition ratio according to the data in the 2020 China Statistical Yearbook. ^Δ^Treatment naïve, without previous corticosteroids or DMARDs treatment for 6 months before recruited. MAFLD, metabolic dysfunction-associated fatty liver disease; NAFLD, non-alcoholic fatty liver disease. remission (CDAI ≤2.8 or DAS28-CRP <2.6); active (CDAI >2.8 or DAS28-CRP ≥2.6); LDA, low disease activity (2.8< CDAI ≤10, 3.3 <SDAI ≤11, 2.6≤ DAS28-CRP <3.2, or 2.6≤ DAS28-ESR <3.2); MDA, moderate disease activity (10< CDAI ≤22, 11< SDAI ≤26, 3.2≤ DAS28-CRP ≤5.1, or 3.2≤ DAS28-ESR ≤5.1); HAD, high disease activity (22< CDAI, 26< SDAI, 5.1< DAS28-CRP, or 5.1< DAS28-ESR).

The prevalence of MAFLD was 21.4% (110/513). Specifically, 19.9% (102/513) patients with RA fulfilled both criteria of MAFLD and NAFLD that were diagnosed as hepatic steatosis without over alcohol intake (≥30 g/d in men and ≥20 g/d in women) or concomitant liver disease (e.g., viral hepatitis, autoimmune liver diseases, etc.) (5). A total of eight (1.6%) RA patients with chronic hepatitis B infection who were not classified as NAFLD were newly identified as MAFLD, whereas 3 (0.6%) RA patients with NAFLD were not complicated with metabolic abnormalities. The prevalence of NAFLD was 20.5% (105/513, Figure 1A).

There was no significant difference in MAFLD prevalence between male and female patients with RA (22.5 vs. 21.1%, *p* = 0.754, Figure 1B). After adjustment for the age and sex composition ratio according to the data in the 2020 China Statistical Yearbook (21), the standardized prevalence of MAFLD in our RA cohort was 20.5%. The men had a higher standardized prevalence of MAFLD (25.4 vs. 15.6%, *p* = 0.020, Figure 1B) than the women.

The prevalence of MAFLD increased with age, and patients with RA at the age of 50–59 years had the highest prevalence (28.5%), followed by 60–69 years (23.3%), and 40–49 years (21.5%, Figure 1C). Patients with RA at the age of 50–59 years had the highest standardized prevalence of MAFLD (27.2%), followed by 40–49 years (25.7%), and 60–69 years (21.0%, Figure 1C). The active patients with RA had a higher prevalence of MAFLD than those in remission (according to CDAI: 23.1 vs. 12.0%, Figure 1D; DAS28-CRP: 23.6 vs. 14.6%, Figure 1E; SDAI: 22.7 vs. 13.9%, Figure 1F and DAS28-ESR: 22.6 vs. 14.1%, Figure 1G), whereas there was no significant difference in MAFLD prevalence among patients with RA with low, moderate, and high disease activity (Figures 1J–M), between treatment naïve and treated patients with RA (Figure 1H), and among different disease durations (Figure 1I).

### Clinical Characteristics in Patients With Rheumatoid Arthritis With Metabolic Dysfunction-Associated Fatty Liver Disease

Patients with RA with MAFLD were older than those without (mean 54.4 vs. 51.1 years, *p* = 0.027), but no significant differences in other RA disease characteristics were observed between the 2 groups (Table 1). As expected, RA patients with MAFLD had a significantly higher prevalence of metabolic abnormalities (Supplementary Table 1). In turn, patients with RA with metabolic abnormalities, including T2DM, elevated TG, overweight or obesity, elevated WC, elevated HOMA-IR, and elevated BP, had a significantly higher prevalence of MAFLD. Notably, RA patients with T2DM had the highest prevalence of MAFLD (43.0%), followed by patients with RA with overweight or obesity (41.1%), and elevated TG (40.9%, Supplementary Figure 1).

Compared with those without, RA patients with MAFLD had slightly elevated ALT (ULN <ALT <2 ULN: 11.8 vs. 4.5%), a higher GGT (median 27 vs. 20), a higher liver fibrosis score (NFS: median −1.56 vs. −2.19; Forns index: median 5.22 vs. 4.89, respectively), and a higher percentage of advanced fibrosis (NFS: 40.9 vs. 23.1%; Forns index: 32.7 vs. 22.8%, all *p* < 0.05, Table 2).

**TABLE 2:** Comparisons of liver biochemistry and fibrosis indices between RA patients with and without MAFLD.

Characteristics	Patients with RA (*n* = 513)	No-MAFLD (*n* = 403)	MAFLD (*n* = 110)	*P*
**Liver biochemistry**				
ALT, UI/L, median (IQR)	16 (10,23)	15 (10,23)	17 (11,27)	0.085
ULN < ALT < 2ULN, n (%)	31 (6.0)	18 (4.5)	13 (11.8)	0.004
2ULN < ALT < 3ULN n (%)	5 (1.0)	3 (0.7)	2 (1.8)	0.292
3ULN < ALT, n (%)	2 (0.4)	2 (0.5)	0 (0)	1.000
AST, UI/L, median (IQR)	18 (14,23)	18 (14,23)	17 (14,21)	0.200
ULN < AST < 2ULN, n (%)	31 (6.0)	22 (5.5)	9 (8.2)	0.288
2ULN < AST < 3ULN, n (%)	2 (0.4)	2 (0.5)	0 (0)	1.000
3ULN < AST, n (%)	2 (0.4)	2 (0.5)	0 (0)	1.000
GGT, UI/L, median (IQR)	22 (15,36)	20 (14,33)	27 (19,44)	<0.001
ULN < GGT < 2 ULN, n (%)	59 (11.5)	42 (10.4)	17 (15.5)	0.143
2ULN < GGT < 3ULN, n (%)	16 (3.1)	12 (3.0)	4 (3.6)	0.757
3ULN < GGT, n (%)	10 (1.9)	6 (1.5)	4 (3.6)	0.233
ALP, UI/L, median (IQR)	81 (65,101)	80 (64,101)	81 (65,102)	0.967
ULN < ALP < 2 ULN, n (%)	32 (6.2)	24 (6.0)	8 (7.3)	0.613
2ULN < ALP < 3ULN, n (%)	0 (0)	0 (0)	0 (0)	-
3ULN < ALP, n (%)	0 (0)	0 (0)	0 (0)	-
TBIL, μmol/L, median (IQR)	8.7 (6.9,10.9)	8.8 (6.9,11.2)	8.2 (6.8,10.6)	0.792
ULN < TBIL < 2 ULN, n (%)	5 (1.0)	4 (1.0)	1 (0.9)	1.000
2ULN < TBIL < 3ULN, n (%)	0 (0)	0 (0)	0 (0)	-
3ULN < TBIL, n (%)	0 (0)	0 (0)	0 (0)	-
ALB, g/L, median (IQR)	34.2 (30.7,37.9)	33.9 (30.4,38.0)	34.4 (31.7,36.9)	0.066
ALB < LLN, n (%)	287 (55.9)	226 (56.1)	61 (55.0)	0.907
Abnormal LFTs, n (%)	118 (23.0)	86 (21.3)	32 (29.1)	0.087
**Hepatic steatosis scores**				
FLI, median (IQR)	11.7 (4.7,25.6)	8.8 (4.0,20.0)	27.1 (13.4,53.2)	<0.001
FLI ≥60, n (%)	17 (3.3)	0 (0)	17 (15.5)	<0.001
**Liver fibrosis score**				
FIB-4, median (IQR)	0.80 (0.54,1.15)	0.80 (0.53,1.15)	0.82 (0.60,1.14)	0.504
Advanced fibrosis, n (%)	70 (13.6)	54 (13.4)	16 (14.5)	0.756
NFS, median (IQR)	−2.12 (−3.27, −1.06)	−2.19 (−3.40, −1.24)	−1.56 (−2.66, −0.38)	<0.001
Advanced fibrosis, n (%)	138 (26.9)	93 (23.1)	45 (40.9)	<0.001
Forns index, median (IQR)	4.92 (3.86,5.88)	4.89 (3.76,5.81)	5.22 (4.26,6.21)	0.008
Advanced fibrosis, n (%)	128 (25.0)	92 (22.8)	36 (32.7)	0.033

*MAFLD, metabolic dysfunction-associated fatty liver disease; ALT, alanine aminotransferase; AST, aspartate aminotransferase; GGT, γ-glutamyl transferase; ALP, alkaline phosphatase; TBIL, total bilirubin; ALB, albumin; ULN, upper limit of normal; LLN, lower limit of normal; LFTs, liver function tests; FLI, fatty liver index; FIB-4, fibrosis-4 index; NFS, NAFLD fibrosis score; IQR, interquartile range.*

### Cardiovascular Disease Events and Estimated 10-Year Cardiovascular Disease Risk in Patients With Rheumatoid Arthritis

In this RA cohort, 56 (10.9%) patients concomitated with CVD events, including ischemic stroke (4.5%), heart failure (3.7%), myocardial infarction (2.3%), angina pectoris (1.4%), and peripheral arterial disease (0.6%). Compared with those without, RA patients with MAFLD had a higher rate of CVD events (17.3 vs. 9.2%), especially higher rates of myocardial infarction (5.5 vs. 1.5%) and angina pectoris (3.6 vs. 0.7%, all *p* < 0.05, Table 3). Patients with RA with MAFLD also had significantly higher estimated 10-year CVD risk scores, including China-PAR, SCORE, and FRS. The median China-PAR was significantly higher in RA patients with MAFLD (3.7 vs. 2.1%). A total of 166 (32.4%) patients with RA had a high estimated 10-year CVD risk. RA patients with MAFLD had a higher proportion of high estimated 10-year CVD risk than those without (55.5 vs. 26.1%, all *p* < 0.05, Table 3).

**TABLE 3:** Comparisons of CVD events and estimated 10-year CVD risk between RA patients with and without MAFLD.

Characteristics	Patients with RA (*n* = 513)	No-MAFLD (*n* = 403)	MAFLD (*n* = 110)	*P*
CVD events, n (%)	56 (10.9)	37 (9.2)	19 (17.3)	0.016
Myocardial infarction, n (%)	12 (2.3)	6 (1.5)	6 (5.5)	0.025
Angina pectoris, n (%)	7 (1.4)	3 (0.7)	4 (3.6)	0.041
Heart failure, n (%)	19 (3.7)	14 (3.5)	5 (4.5)	0.574
Ischemic stroke, n (%)	23 (4.5)	18 (4.5)	5 (4.5)	1.000
Hemorrhagic stroke, n (%)	0 (0)	0 (0)	0 (0)	-
Peripheral arterial disease, n (%)	3 (0.6)	1 (0.2)	2 (1.8)	0.118
**Estimated 10-year CVD risk**				
SCORE for low-risk region, median (IQR)	0.0 (0.0,3.0)	0.0 (0.0,1.5)	1.5 (0.0,3.0)	0.023
SCORE for high-risk region, median (IQR)	1.5 (0.0,4.5)	1.5 (0.0,4.5)	1.5 (1.5,4.5)	0.003
FRS, median (IQR)	1.5 (0.0,7.5)	1.5 (0.0,7.5)	3.0 (1.5,6.0)	0.002
China-PAR equation, median (IQR)	2.5 (0.9,6.0)	2.1 (0.8,5.8)	3.7 (2.0,7.7)	<0.001
High estimated 10-year CVD risk, n (%)	166 (32.4)	105 (26.1)	61 (55.5)	<0.001

*MAFLD, metabolic dysfunction-associated fatty liver disease; CVD, cardiovascular disease; SCORE, the Systemic Coronary Risk Evaluation; FRS, Framingham Risk Score; China-PAR, the Prediction for ASCVD Risk in China; IQR, interquartile range.*

Compared with those without CVD, RA patients with CVD were older (mean 62.6 vs. 50.5 years), having more severe RA disease (Supplementary Table 2), higher WC (mean 83.9 vs. 79.0 cm), and LDL-C (mean 3.40 vs. 3.05 mmol/L), as well as higher prevalence of hypertension (57.1 vs. 30.0%), T2DM (32.1 vs. 13.3%), and AF (8.9 vs. 0.9%, all *p* < 0.05, Supplementary Table 3). In addition, RA patients with CVD had a higher percentage of MAFLD (33.9 vs. 19.9%), as well as a higher percentage of abnormal LFTs (33.9 vs. 21.7%), higher hepatic steatosis score (FLI: median 19.0 vs. 10.8), liver fibrosis scores (FIB-4: median 0.94 vs. 0.79; NFS: median −1.64 vs. −2.15; Forns index: median 5.54 vs. 4.86), and a higher percentage of advanced fibrosis (defined by Forns index: 39.3 vs. 23.2%, all *p* < 0.05, Supplementary Table 4).

### Factors Associated With Cardiovascular Disease Events and High Estimated 10-Year Cardiovascular Disease Risk in Patients With Rheumatoid Arthritis

**FIGURE 2:**
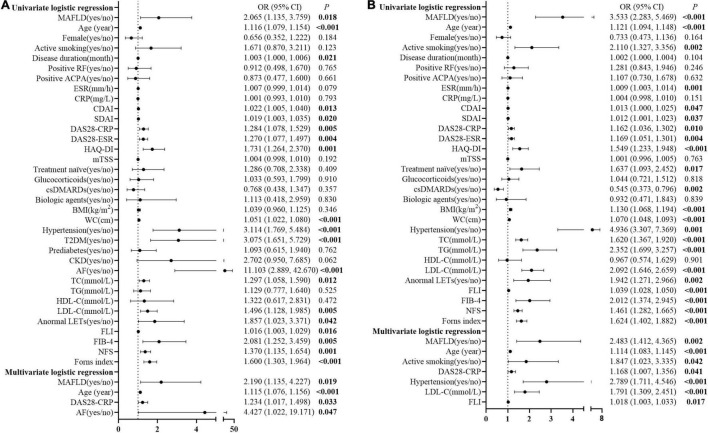
Logistic regression analysis for potential associated factors of CVD events and high estimated 10-year CVD risk in patients with RA. Forest plots show OR and 95% CI for CVD events **(A)** and high estimated 10-year CVD risk **(B)** in patients with RA. MAFLD, metabolic dysfunction-associated fatty liver disease; RF, rheumatoid factor; ACPA, anti-cyclic citrullinated peptide antibody; ESR, erythrocyte sedimentation rate; CRP, C-reactive protein; CDAI, clinical disease activity index; SDAI, simplified disease activity index; DAS28-CRP, disease activity score in 28 joints with four variables, including CRP; DAS28-ESR, disease activity score in 28 joints with four variables including ESR; HAQ-DI, health assessment questionnaire disability index; mTSS, modified total Sharp score; csDMARD, conventional synthetic disease-modifying anti-rheumatic drug; BMI, body mass index; WC, waist circumference; T2DM, type 2 diabetes mellitus; TC, total cholesterol; TG, triglyceride; HDL-C, high-density lipoprotein cholesterol; LDL-C, low-density lipoprotein cholesterol; LFTs, liver function tests; FLI, fatty liver index; FIB-4, fibrosis-4 index; NFS, NAFLD fibrosis score; OR, odds ratio; CI, confidence interval.

As shown in Figure 2, univariate logistic regression analysis showed that MAFLD, age, disease duration, CDAI, SDAI, DAS28-CRP, DAS28-ESR, HAQ-DI, WC, hypertension, T2DM, AF, TC, LDL-C, anormal LETs, FLI, FIB-4, NFS, and Forns index were associated with CVD events in patients with RA. Further stepwise multivariate logistic regression analysis, including the above-mentioned significant indicators and the established risk factors (including sex and active smoking), showed that MAFLD [adjusted OR (AOR) = 2.190, 95% CI 1.135–4.227], age (AOR = 1.115, 95% CI 1.076–1.156), DAS28-CRP (AOR = 1.234, 95% CI 1.017–1.498), and AF (AOR = 4.427, 95% CI 1.022–19.171, all *p* < 0.05, Figure 2A) were associated with an increase in CVD events in patients with RA.

Moreover, MAFLD, age, active smoking, ESR, CDAI, SDAI, DAS28-CRP, DAS28-ESR, HAQ-DI, treatment naïve, conventional synthetic disease-modifying anti-rheumatic drugs (csDMARDs), BMI, WC, hypertension, TC, TG, LDL-C, anormal LETs, FLI, FIB-4, NFS, and Forns index were associated with the high estimated 10-year CVD risk for patients with RA in the unadjusted univariate logistic regression analysis. After adjustment for the potential confounders, including sex and the above-mentioned significant indicators, MAFLD (AOR = 2.483, 95% CI 1.412–4.365), age (AOR = 1.114, 95% CI 1.083–1.145), active smoking (AOR = 1.847, 95% CI 1.023–3.335), DAS28-CRP (AOR = 1.168, 95% CI 1.007–1.356), hypertension (AOR = 2.789, 95% CI 1.711–4.546), LDL-C (AOR = 1.791, 95% CI 1.309–2.451), and FLI (AOR = 1.018, 95% CI 1.003–1.033, all *p* < 0.05, Figure 2B) were the associated factors with high estimated 10-year CVD risk in patients with RA.

## Discussion

Our research for the first reports that the prevalence of MAFLD in patients with RA is 21.4%, which is similar to that of NAFLD. Besides a higher risk of advanced fibrosis, MAFLD is associated with an increase in CVD events (2–3-folds) and a high estimated 10-year CVD risk. These findings impose an emphasis on the detection and management of MAFLD in patients with RA.

A recent meta-analysis in 2020 reported the total prevalence of NAFLD in Mainland China is 29.9%, which was similar to that in western countries (23.7–31.8%) (22, 23). In addition, the epidemiological data on MAFLD prevalence were 30–40% in the general population in Asia (24). Although the definition of MAFLD allowed the inclusion of patients with over alcohol intake and concomitant liver diseases, the dispute remained on whether more patients were identified by using the MAFLD definition. In a Korea Nationwide Cohort Study of 9.58 million middle-aged adults, the prevalence of fatty liver disease (FLD) was higher using the definition of MAFLD than NAFLD (37.3 vs. 28.0%) (25). A population-based study in northern China also showed a higher prevalence of MAFLD than NAFLD (32.7 vs. 27.3%) (26). However, other researchers showed an identical prevalence between MAFLD and NAFLD (31.2 vs. 33.2%) by Lin et al. (27), 25.9 vs. 25.7% by Wong et al. (28), and 27.4 vs. 27.9% by Huang et al. (29). In our cohort, the prevalence of NAFLD was 20.5%, which was consistent with that in the Korean (21.1%) and in the Italian (25.0%) RA population (30, 31). To our best knowledge, no studies have investigated the epidemiological data of MAFLD in patients with RA yet. In this study, the prevalence of MAFLD was 21.4%, which indicated the new definition of MAFLD shared a similar prevalence to NAFLD in patients with RA. The presence of metabolic dysfunction is mainly responsible for MAFLD (7). However, in this study, the rate of overweight or obesity, one of the key metabolic traits in MAFLD, in Chinese patients with RA was 32.7%, which was lower than that in patients with RA from western countries, such as Germany (57.0–62.6%) (32) or the US (62.6%) (33), and in Chinese adults (50.7%) (34). Meanwhile, patients with RA have a lower lipid level than the general population, which is associated with inflammation in these patients (3). For example, a study from Swedish including 1,179 RA and 4,78,627 non-RA persons revealed that patients with RA have lower TC and LDL-C than non-RA persons (35). A meta-analysis, including 53 studies, reported that the rate of high TG, another key characteristic of a metabolic trait in MAFLD, in patients with RA was 35.4% (36). The rate of high TG in Chinese adults was 25.8% (37). In our study, the rate of high TG in Chinese patients with RA was as low as 12.9%. A lower rate of overweight or obesity and a lower rate of high TG in our study of Chinese patients with RA may be the possible reason for no more patients identified using the new MAFLD definition.

Reports from the general population have demonstrated that individuals with MAFLD have a higher level of liver parameters (ALT, AST, ALP, and GGT) and more proportion of abnormal ALT than those without (25, 26). Patients with MAFLD showed a higher liver fibrosis score and a higher ratio of advanced fibrosis than the general population (27, 28). Moreover, the sensitivity for detecting advanced fibrosis was higher for MAFLD than NAFLD (93.9 vs. 73.0%), in which the MAFLD definition better identifies advanced fibrosis in the general population (38). In our study, RA patients with MAFLD showed slightly elevated ALT, higher liver fibrosis scores, and advanced fibrosis. As recommended by the 2021 ACR Guideline for RA treatment (39), clinicians should pay attention to NAFLD (or MAFLD) and dynamically monitor liver enzymes, liver function tests, and liver fibrosis when using methotrexate and other hepatic toxic medicines.

Cardiovascular disease is the primary reason for deaths among patients with RA. Previous research reported that patients with RA were more likely to have a history of myocardial infarction (3.1 vs. 2.6%), heart failure (1.6 vs. 1.0%), or stroke (3.9 vs. 2.7%) at diagnosis, and a higher number of CVD events occurred (events per 1,000 person-years 10.6 vs. 8.1) in the first 5 years than controls (40). In our RA cohort, 10.9% (56/513) patients concomitated with CVD events, which was similar to a large-scale multicenter survey of 21 hospitals in China (12.7%, 256/2013) (41), although a comprehensive meta-analysis demonstrated that NAFLD conferred an OR of 1.64 for fatal and/or non-fatal CVD events (42). Recently, a large-scale matched cohort research including 17.7 million participants showed that NAFLD was not significantly related to stroke or acute myocardial infarction risk after adjustment for established CVD risk factors (43). In this concern, the alteration from NAFLD to MAFLD was proposed for identifying more patients at an increased risk of CVD in the general population (25, 44). Several studies showed that MAFLD was related to an increased hazard of CVD than those without 1.43-folds by Lee et al. (25) and 1.89-folds by Yoneda et al. (45). These data indicated that both NAFLD and MAFLD were the independent risk factors for CVD in the general population. There are multiple underlying mechanisms by which FLD increases the risk of CVD, such as systemic inflammation and oxidative stress, hepatic insulin resistance, platelet activation, endothelial dysfunction, and so on (6). In this study, we first reported that RA patients with MAFLD were associated with an increased CVD risk, including CVD events (AOR = 2.218) and a high estimated 10-year CVD risk (AOR = 2.483). According to these data, all RA patients with MAFLD should undergo careful CVD surveillance as recommended by the American Association for the Study for Liver Diseases (AASLD) guidelines for all patients with MAFLD or NAFLD, either by Framingham Risk Score, SCORE, or by other risk charts for CVD risk assessments (46, 47).

Type 2 diabetes mellitus and IR are the most important risk factors for NAFLD or MAFLD development, which strongly predict adverse clinical outcomes, such as advanced hepatic fibrosis and mortality (48, 49). In turn, fat accumulation in the liver causes hepatic IR, boosts increased glucose production in the liver, and in turn acts as a further stimulus for increasing whole-body IR (50) and CVD risks (51). MAFLD (defined by hepatic steatosis index >36) was present in 76.3% of the 78,895 patients with T2DM in Italian specialist care (52). Diabetes MAFLD had more significant fibrosis than overweight, obese, lean or normal weight MAFLD (18.9 vs. 1.3% vs. 5.5 vs. 6.4%) (52). In this study, the prevalence of T2DM in RA patients with MAFLD was 30.9%, and RA patients with T2DM had a higher prevalence of MAFLD than non-T2DM patients with RA (43.0 vs. 17.5%), similar to that in the general population (47.2 vs. 25.9%) (53).

There were several potential limitations in this study. First, ultrasound, rather than liver biopsy, was used to identify hepatic steatosis. However, one qualified meta-analysis showed high sensitivity and specificity in the detection of moderate-severe hepatic steatosis by ultrasound (54). Second, CRP rather than hs-CRP was regarded as one of the indicators of metabolic abnormalities in lean or normal weight MAFLD definition in our study. However, the remaining 3 patients who were not diagnosed with MAFLD had neither T2DM, overweight/obesity, nor any other metabolic disorders than CRP. Thus, the prevalence of MAFLD was not affected by CRP in our study. Third, as for the nature of any observational studies, we cannot exclude the possibility of residual confounding completely despite our careful adjustment for the potential risk factors.

## Conclusion

Metabolic dysfunction-associated fatty liver disease is a common comorbidity in patients with RA and is associated with increased CVD risk. These data imply the importance of MAFLD detection and dynamic monitoring in patients with RA, especially with T2DM or overweight or obesity. All RA patients with MAFLD should undergo careful cardiovascular surveillance and primary prevention, and treatments to modify MAFLD also need consideration in the management of RA.

## Data Availability Statement

The raw data supporting the conclusions of this article will be made available by the authors, without undue reservation.

## Ethics Statement

The studies involving human participants were reviewed and approved by Ethics Committee at Sun Yat-sen Memorial Hospital (SYSEC-KY-KS-2020-208). The patients/participants provided their written informed consent to participate in this study.

## Author Contributions

Y-WZ and Q-HL contributed equally to this work, including conceiving and designing the study, reading and analyzing documents, performing statistical analysis, and drafting the manuscript. J-WG participated in analyzing documents and drafting the manuscript. JP and L-FC participated in the clinical assessment. J-DM and J-ZL carried out the radiographic assessment. Y-QM and X-PZ participated in data collection. P-ML and LD conceived and participated in its design, read and analyzed documents, and edited the manuscript. All authors read and approved the final manuscript.

## Conflict of Interest

The authors declare that the research was conducted in the absence of any commercial or financial relationships that could be construed as a potential conflict of interest.

## Publisher’s Note

All claims expressed in this article are solely those of the authors and do not necessarily represent those of their affiliated organizations, or those of the publisher, the editors and the reviewers. Any product that may be evaluated in this article, or claim that may be made by its manufacturer, is not guaranteed or endorsed by the publisher.
