# Supplementary material for: Association Between Metabolic Dysfunction-Associated Fatty Liver Disease and Cardiovascular Risk in Patients With Rheumatoid Arthritis: A Cross-Sectional Study of Chinese Cohort
Source: Front Cardiovasc Med. 2022 May 13;9:884636. doi: 10.3389/fcvm.2022.884636 (PMC9136028; doi:10.3389/fcvm.2022.884636)
Supplement: Supplementary file 1 [file Data_Sheet_1.docx]

**Supplementary Materials**

**Supplementary Table S1 The prevalence of metabolic abnormalities in RA patients with MAFLD**

| **Characteristics** | **RA patients**  **(n=513)** | **No-MAFLD**  **(n=403)** | **MAFLD**  **(n=110)** | ***P*** |
| --- | --- | --- | --- | --- |
| BMI, kg/m^2^, mean ± SD | 21.7±3.4 | 21.1±3.0 | 24.1±3.7 | **<0.001** |
| WC, cm, mean ± SD | 79.5±9.7 | 77.6±8.8 | 86.3±10.1 | **<0.001** |
| Overweight/obese, n (%) | 168 (32.7) | 99 (24.6) | 69 (62.7) | **<0.001** |
| SBP, mmHg, mean ± SD | 125.7±20.0 | 124.4±19.9 | 130.4±19.8 | **0.005** |
| DBP, mmHg, mean ± SD | 78.8±11.3 | 78.3±11.0 | 80.8±11.9 | **0.044** |
| Hypertension, n (%) | 169 (32.9) | 117 (29.0) | 52 (47.3) | **<0.001** |
| HbA1C%, mean ± SD | 5.67±1.04 | 5.56±0.82 | 6.10±1.54 | **<0.001** |
| FPG, mmol/L, mean ± SD | 5.62±2.19 | 5.52±2.11 | 5.95±2.46 | 0.095 |
| FINS, IU/mL, mean ± SD | 9.39±5.75 | 8.74±5.14 | 11.80±7.09 | **<0.001** |
| HOMA-IR, mean ± SD | 2.46±2.50 | 2.26±2.30 | 3.22±3.01 | **0.002** |
| Prediabetes, *n* (%) | 183 (35.7) | 145 (36.0) | 38 (34.5) | 0.781 |
| T2DM, *n* (%) | 79 (15.4) | 45 (11.2) | 34 (30.9) | **<0.001** |
| TC, mmol/L, mean ± SD | 4.90±1.22 | 4.77±1.10 | 5.36±1.48 | **<0.001** |
| TG, mmol/L, mean ± SD | 1.12±0.68 | 1.03±0.52 | 1.47±1.00 | **<0.001** |
| Elevated TG, *n* (%) | 66 (12.9) | 39 (9.7) | 27 (24.5) | **<0.001** |
| HDL-C, mmol/L, mean ± SD | 1.32±0.36 | 1.31±0.35 | 1.34±0.36 | 0.440 |
| Reduced HDL-C, *n* (%) | 139 (27.1) | 115 (28.9) | 24 (21.8) | 0.160 |
| LDL-C, mmol/L, mean ± SD | 3.09±0.88 | 3.00±0.79 | 3.40±1.07 | **<0.001** |
| eGFR, ml/min/1.73 m^2^, mean ± SD | 103±29 | 105±31 | 96±21 | **<0.001** |
| CKD, *n* (%) | 21 (4.1) | 16 (4.0) | 5 (4.5) | 0.787 |
| AF, *n* (%) | 9 (1.8) | 6 (1.5) | 2 (2.7) | 0.412 |

Abbreviations: MAFLD, metabolic dysfunction-associated fatty liver disease; BMI, body mass index; WC, waist circumference; SBP, systolic blood pressure; DBP, diastolic blood pressure; HbA1C, hemoglobin A1c; FPG, fasting plasma glucose; FINS, fasting insulin; TC, total cholesterol; TG, triglyceride; HDL-C, high-density lipoprotein cholesterol; LDL-C, low-density lipoprotein cholesterol; eGFR, estimated glomerular filtration rate; CKD, chronic kidney disease; AF, atrial fibrillation; SD, standard deviations

**Supplementary Table S2 Demographic and clinical characteristics of RA patients with CVD**

| **Characteristics** | **RA patients**  **(n=513)** | **No-CVD**  **(n=457)** | **CVD**  **(n=56)** | ***P*** |
| --- | --- | --- | --- | --- |
| Age, years, mean ± SD | 51.8±12.6 | 50.5±12.4 | 62.6±8.3 | **<0.001** |
| Female, *n* (%) | 402 (78.4) | 362 (79.2) | 40 (71.4) | 0.182 |
| Active smoking, *n* (%) | 90 (17.5) | 76 (16.6) | 14 (25.0) | 0.120 |
| Disease duration, months, median (IQR) | 60 (21,120) | 58 (21,120) | 78 (15,179) | 0.247 |
| Positive RF, *n* (%) | 366 (71.3) | 327 (71.6) | 39 (69.6) | 0.765 |
| Positive ACPA, *n* (%) | 370 (72.1) | 331 (72.4) | 39 (69.6) | 0.661 |
| Core disease activity indicators |  |  |  |  |
| 28TJC, median (IQR) | 4 (1,9) | 4 (1,9) | 5 (1,12) | 0.099 |
| 28SJC, median (IQR) | 2 (0,7) | 2 (0,6) | 3 (1,9) | 0.091 |
| PtGA, cm, median (IQR) | 4 (2,6) | 4 (2,6) | 6 (4,8) | **<0.001** |
| PrGA, cm, median (IQR) | 4 (2,6) | 4 (2,6) | 6 (4,8) | **<0.001** |
| Pain VAS, cm, median (IQR) | 4 (2,5) | 4 (2,5) | 4 (2,6) | **0.046** |
| ESR, mm/h, median (IQR) | 39 (21,70) | 36 (20,69) | 50 (33,76) | **0.009** |
| CRP, mg/L, median (IQR) | 7.7 (3.3,30.4) | 7.2 (3.3,29.9) | 11.9 (3.3,37.4) | 0.146 |
| CDAI, median (IQR) | 16 (6,28) | 14 (6,26) | 22 (12,36) | **0.004** |
| SDAI, median (IQR) | 17.3 (7.3,31.9) | 16.7 (6.3,30.4) | 24.8 (13.5,38.1) | **0.006** |
| DAS28-CRP, median (IQR) | 4.0 (2.8,5.4) | 3.9 (2.6,5.3) | 4.9 (3.4,5.9) | **0.003** |
| DAS28-ESR, median (IQR) | 4.7 (3.2,6.1) | 4.6 (3.0,6.0) | 5.5 (3.9,6.5) | **0.004** |
| Functional indicator |  |  |  |  |
| HAQ-DI, median (IQR) | 0.50 (0.00,1.13) | 0.38 (0.00,1.13) | 1.00 (0.38,1.75) | **<0.001** |
| Radiographic indicators |  |  |  |  |
| mTSS, median (IQR) | 9 (1,34) | 9 (1,31) | 13 (4,51) | **0.029** |
| JSN, median (IQR) | 2 (0,15) | 2 (0,14) | 2 (0,23) | 0.459 |
| JE, median (IQR) | 6 (0,19) | 6 (0,17) | 11 (3,29) | **0.008** |
| Previous medications |  |  |  |  |
| Treatment naïve^△^, *n* (%) | 141 (27.5) | 123 (26.9) | 18 (32.1) | 0.408 |
| Glucocorticoid, *n* (%) | 262 (51.1) | 233 (51.0) | 29 (51.8) | 0.910 |
| csDMARDs, n (%) | 322 (62.8) | 290 (63.5) | 32 (57.1) | 0.356 |
| Biologic agents, *n* (%) | 42 (8.2) | 37 (8.1) | 5 (8.9) | 0.830 |

^△^Treatment naïve, without previous corticosteroids or DMARDs treatment for six months before recruited.

Abbreviations: MAFLD, metabolic dysfunction-associated fatty liver disease; RF, rheumatoid factor; ACPA, anti-cyclic citrullinated peptide antibody; 28TJC, 28-joint tender joint counts; 28SJC, 28-joint swollen joint counts; PtGA, patient global assessment of disease activity; PrGA, provider global assessment of disease activity; Pain VAS, pain visual analogue scale; ESR, erythrocyte sedimentation rate; CRP, C reactive protein; CDAI, clinical disease activity index; HAQ-DI, health assessment questionnaire disability index; mTSS, modified total Sharp score; JE, joint erosion; JSN, joint space narrowing; csDMARDs, conventional synthetic disease-modifying anti-rheumatic drugs; SD, standard deviations; IQR, interquartile range.

**Supplementary Table S3 The prevalence of metabolic abnormalities in RA patients with CVD**

| **Characteristics** | **RA patients**  **(n=513)** | **No-CVD**  **(n=457)** | **CVD**  **(n=56)** | ***P*** |
| --- | --- | --- | --- | --- |
| BMI, kg/m^2^, mean ± SD | 21.7±3.4 | 21.7±3.4 | 22.1±3.5 | 0.346 |
| WC, cm, mean ± SD | 79.5±9.7 | 79.0±9.5 | 83.9±10.7 | **<0.001** |
| Overweight/obese, n (%) | 168 (32.7) | 145 (31.7) | 23 (41.1) | 0.160 |
| SBP, mmHg, mean ± SD | 125.7±20.0 | 124.6±19.6 | 134.4±21.7 | **0.001** |
| DBP, mmHg, mean ± SD | 78.8±11.3 | 78.6±11.0 | 80.9±13.3 | 0.141 |
| Hypertension, *n* (%) | 169 (32.9) | 137 (30.0) | 32 (57.1) | **<0.001** |
| HbA1C%, mean ± SD | 5.67±1.04 | 5.64±1.04 | 5.98±0.98 | **0.019** |
| FPG, mmol/L, mean ± SD | 5.62±2.19 | 5.57±2.21 | 5.96±2.09 | 0.211 |
| FINS, IU/mL, mean ± SD | 9.39±5.75 | 9.26±5.39 | 10.48±8.09 | 0.279 |
| HOMA-IR, mean ± SD | 2.46±2.50 | 2.40±2.38 | 3.00±3.33 | 0.191 |
| T2DM, *n* (%) | 79 (15.4) | 61 (13.3) | 18 (32.1) | **<0.001** |
| Prediabetes, *n* (%) | 183 (35.7) | 162 (35.4) | 21 (37.5) | 0.762 |
| TC, mmol/L, mean ± SD | 4.90±1.22 | 4.85±1.14 | 5.29±1.68 | 0.062 |
| TG, mmol/L, mean ± SD | 1.12±0.68 | 1.12±0.69 | 1.18±0.55 | 0.524 |
| Elevated TG, *n* (%) | 66 (12.9) | 56 (12.3) | 10 (17.9) | 0.237 |
| HDL-C, mmol/L, mean ± SD | 1.32±0.36 | 1.31±0.35 | 1.35±0.42 | 0.473 |
| Reduced HDL-C, *n* (%) | 139 (27.1) | 121 (26.5) | 18 (32.1) | 0.368 |
| LDL-C, mmol/L, mean ± SD | 3.09±0.88 | 3.05±0.82 | 3.40±1.20 | **0.036** |
| eGFR, ml/min/1.73 m^2^, mean ± SD | 103±29 | 104±29 | 97±33 | 0.093 |
| CKD, *n* (%) | 21 (4.1) | 16 (3.5) | 5 (8.9) | 0.067 |
| AF, *n* (%) | 9 (1.8) | 4 (0.9) | 5 (8.9) | **0.001** |

Abbreviations: MAFLD, metabolic dysfunction-associated fatty liver disease; BMI, body mass index; WC, waist circumference; SBP, systolic blood pressure; DBP, diastolic blood pressure; HbA1C, hemoglobin A1c; FPG, fasting plasma glucose; FINS, fasting insulin; TC, total cholesterol; TG, triglyceride; HDL-C, high-density lipoprotein cholesterol; LDL-C, low-density lipoprotein cholesterol; eGFR, estimated glomerular filtration rate; CKD, chronic kidney disease; AF, atrial fibrillation; SD, standard deviations

**Supplementary Table S4 Comparisons of liver biochemistry and fibrosis indices between RA patients with and without CVD**

| **Characteristics** | **RA patients**  **(n=513)** | **No-CVD**  **(n=457)** | **CVD**  **(n=56)** | ***P*** |
| --- | --- | --- | --- | --- |
| MAFLD, n (%) | 110 (21.4) | 91 (19.9) | 19 (33.9) | **0.016** |
| **Liver biochemistry** |  |  |  |  |
| ALT, UI/L, median (IQR) | 16 (10,23) | 15 (10,23) | 18 (14,26) | **0.038** |
| ULN <ALT<2ULN, n (%) | 31 (6.0) | 26 (5.7) | 5 (8.9) | 0.367 |
| 2ULN<ALT<3ULN n (%) | 5 (1.0) | 5 (1.1) | 0 (0) | 1.000 |
| 3ULN<ALT, n (%) | 2 (0.4) | 2 (0.4) | 0 (0) | 1.000 |
| AST, UI/L, median (IQR) | 18 (14,23) | 18 (14,22) | 18 (14,25) | 0.285 |
| ULN <AST<2ULN, n (%) | 31 (6.0) | 27 (5.9) | 4 (7.1) | 0.764 |
| 2ULN<AST<3ULN, n (%) | 2 (0.4) | 2 (0.4) | 0 (0) | 1.000 |
| 3ULN<AST, n (%) | 2 (0.4) | 2 (0.4) | 0 (0) | 1.000 |
| GGT, UI/L, median (IQR) | 22 (15,36) | 21 (15,33) | 30 (19,56) | **0.005** |
| ULN <GGT<2 ULN, n (%) | 59 (11.5) | 43 (9.4) | 16 (28.6) | **<0.001** |
| 2ULN<GGT<3ULN, n (%) | 16 (3.1) | 16 (3.5) | 0 (0) | 0.238 |
| 3ULN<GGT, n (%) | 10 (1.9) | 10 (2.2) | 0 (0) | 0.611 |
| ALP, UI/L, median (IQR) | 81 (65,101) | 80 (64,101) | 84 (66,110) | 0.418 |
| ULN <ALP<2 ULN, n (%) | 32 (6.2) | 27 (5.9) | 5 (8.9) | 0.377 |
| 2ULN<ALP<3ULN, n (%) | 0 (0) | 0 (0) | 0 (0) | - |
| 3ULN<ALP, n (%) | 0 (0) | 0 (0) | 0 (0) | - |
| TBIL, μmol/L, median (IQR) | 8.7 (6.9,10.9) | 8.6 (6.9,10.9) | 8.9 (6.6,11.0) | 0.803 |
| ULN<TBIL<2 ULN, n (%) | 5 (1.0) | 4 (0.9) | 1 (1.8) | 0.440 |
| 2ULN<TBIL<3ULN, n (%) | 0 (0) | 0 (0) | 0 (0) | - |
| 3ULN <TBIL, n (%) | 0 (0) | 0 (0) | 0 (0) | - |
| ALB, g/L, median (IQR) | 34.2 (30.7,37.9) | 34.3 (30.9,38.0) | 33.0 (29.8,36.1) | 0.084 |
| ALB<LLN, n (%) | 287 (55.9) | 251 (54.9) | 36 (64.3) | 0.183 |
| Abnormal LFTs, n (%) | 118 (23.0) | 99 (21.7) | 19 (33.9) | **0.040** |
| **Hepatic steatosis scores** |  |  |  |  |
| FLI, median (IQR) | 11.7 (4.7,25.6) | 10.8 (4.5,24.5) | 19.0 (8.0,33.9) | **0.003** |
| FLI≥60, n (%) | 17 (3.3) | 12 (2.6) | 5 (8.9) | **0.013** |
| **Liver fibrosis score** |  |  |  |  |
| FIB-4, median (IQR) | 0.80 (0.54,1.15) | 0.79 (0.52,1.13) | 0.94 (0.77,1.21) | **0.003** |
| Advanced fibrosis, n (%) | 70 (13.6) | 61 (13.3) | 9 (16.1) | 0.575 |
| NFS, median (IQR) | -2.12 (-3.27,-1.06) | -2.15 (-3.34,-1.11) | -1.64 (-2.52,-0.49) | **0.002** |
| Advanced fibrosis, n (%) | 138 (26.9) | 124 (27.1) | 14 (25.0) | 0.734 |
| Forns index, median (IQR) | 4.92 (3.86,5.88) | 4.86 (3.78,5.81) | 5.54 (4.84,6.47) | **<0.001** |
| Advanced fibrosis, n (%) | 128 (25.0) | 106 (23.2) | 22 (39.3) | **0.009** |

Abbreviations: MAFLD, metabolic dysfunction-associated fatty liver disease; ALT, alanine aminotransferase; AST, aspartate aminotransferase; GGT, γ-glutamyl transferase; ALP, alkaline phosphatase; TBIL, total bilirubin; ALB, albumin; ULN, upper limit of normal; LLN, lower limit of normal; LFTs, liver function tests; FLI, fatty liver index; FIB-4, fibrosis-4 index; NFS, NAFLD fibrosis score; IQR, interquartile range.

**Supplementary Figure S1 The prevalence of MAFLD in RA patients with different metabolic abnormalities**

Abbreviations: MAFLD, metabolic dysfunction-associated fatty liver disease; TG, triglyceride; T2DM, type 2 diabetes mellitus; WC, waist circumference; HOMA-IR, homeostasis model assessment of insulin resistance; BP, blood pressure; HDL-C, high-density lipoprotein cholesterol; CRP, C reactive protein; *** *p* < 0.001.
